# The Efficacy and Safety of Patiromer for Heart Failure Patients: A Systematic Review and Meta-Analysis

**DOI:** 10.1007/s10557-023-07473-w

**Published:** 2023-06-07

**Authors:** Yuhui Wang, Yu Gao, Jun Feng, Linlin Hou, Chunmiao Luo, Zhipeng Zhang

**Affiliations:** 1 Hefei Hospital Affiliated to Anhui Medical University, The Second People’s Hospital of Hefei, Hefei, People’s Republic of China; 2 The Fifth Clinical College of Anhui Medical University, Hefei, People’s Republic of China

**Keywords:** Patiromer, Hyperkalemia, Heart failure, RAASi

## Abstract

**Purpose:**

To evaluate the efficacy and safety of patiromer, a novel potassium binder, in reducing the risk of hyperkalemia in patients with heart failure and optimizing their RAASi therapy.

**Design:**

Systematic review and meta-analyses.

**Method:**

The authors conducted a systematic search in Pubmed, Embase, Web of Science, and Cochrane Library for randomized controlled trials investigating the efficacy and safety of patiromer in heart failure patients from inception to 31 January 2023 and updated on 25 March 2023. The primary outcome was the association between the reduction of hyperkalemia and patiromer compared with placebo, and the secondary outcome was the association between optimization of RAASi therapy and patiromer.

**Results:**

A total of four randomized controlled trials (*n* = 1163) were included in the study. Patiromer was able to reduce the risk of hyperkalemia in heart failure patients by 44% (RR 0.56, 95% CI 0.36 to 0.87; I^2^ = 61.9%), improve tolerance to target doses of MRA in patients with heart failure (RR 1.15, 95% CI 1.02 to 1.30; I^2^ = 49.4%), and decrease the proportion of all-cause discontinuation of RAASi (RR 0.49, 95% CI 0.25 to 0.98; I^2^ = 48.4%). However, patiromer therapy was associated with an increased risk of hypokalemia (RR 1.51, 95% CI 1.07 to 2.12; I^2^ = 0%), while no other statistically significant adverse events were observed.

**Conclusion:**

Patiromer appears to have a considerable effect on reducing the incidence of hyperkalemia in heart failure patients and on optimizing the therapy of RAASi in those patients.

**Supplementary Information:**

The online version contains supplementary material available at 10.1007/s10557-023-07473-w.

## Introduction

Heart failure (HF) is a global pandemic, affecting up to 37.7 million people worldwide, with a prevalence of approximately 1–2% in the adult population in developed countries, rising to over 10% in people over 80 years of age [1]. In the case of HF, disturbances in potassium homeostasis are rather common [2]. According to a recent large observational study, 24.4% of heart failure patients experienced at least one hyperkalemia event within one year, and 10.2% reported moderate or severe hyperkalemia [3].

Studies have shown that hyperkalemia is associated with an increased risk of mortality and other adverse events in HF patients, including those with heart failure with reduced ejection fraction (HFrEF) and heart failure with preserved ejection fraction (HFpEF) [4–9].

Renin-angiotensin-aldosterone system inhibitors (RAASi) are first-line therapies for preventing the progression of cardiovascular disease [10]. However, these drugs often have to be reduced or discontinued due to the induction of hyperkalemia, which prevents some patients from benefiting from these therapies [11–13].

Patiromer is a novel potassium binder that can exchange potassium (K^+^) for calcium (Ca^2+^) in the gastrointestinal tract and can be used to improve the control of serum potassium [14].

2022ACC/AHA/HFSA guidelines expounded that the effectiveness of patiromer to improve outcomes of heart failure patients by facilitating the continuation of RAASi therapy is uncertain. The class of recommendation (COR) was 2b, and the level of evidence (LOE) was B-R [10].

Several randomized controlled trials (RCTs) have investigated and reported the effect of patiromer in lowering mean serum potassium levels [ 15–18], reducing the incidence of hyperkalemia, and optimizing RAASi therapy in HF patients. However, the reported outcomes remain inconclusive.

A recent meta-analysis reported the efficacy of novel potassium binders, including patiromer and SZC, to optimize the RAASi therapy in HF patients [19]. However, there is currently no available meta-analysis specifically focused on patiromer as a single drug, assessing its efficacy of reducing the incidence of hyperkalemia, increasing the tolerance of target dose of MRA, and decreasing discontinuation of RAASi therapy in HF patients. Considering the distinctions in pharmacokinetic, pharmacodynamic, and safety profile respects between patiromer and sodium zirconium cyclosilicate (SZC), only studies that compared patiromer with placebo were included in this study.

Considering the factors mentioned above, we conducted a systematic review and meta-analysis of existing evidence from RCTs to quantitatively evaluate the potential of this drug.

## Methods

We followed a guide on how to design, conduct, and publish a systematic review and meta-analysis. Reporting was done in accordance with PRISMA (*Preferred Reporting Items for Systematic Reviews and Meta-Analyses*) guidelines [20]. We registered the protocol for this systematic review with PROSPERO (CRD42023395789).

### Data Sources and Searches

The literature search was conducted in Pubmed, Embase, Web of Science, and Cochrane Library from inception to January 31 for potentially relevant studies, and the search was updated on 25 March 2023. Supplementary Appendix S1 provides full details of the search strategy.

### Study Selection

We considered studies eligible for inclusion if they: (1) were RCTs, (2) involved HF patients, (3) examined the effects of patiromer on reducing hyperkalemia or optimizing RAASi therapy, and (4) compared patiromer with placebo. The exclusion criteria were as follows: (1) animal experiments or (2) repeated studies. Study selection was performed with two phases: primary screening of title and abstract, then full-text review for potentially eligible articles. Two review authors (L.H. and Y.G.) independently evaluated eligibility, with discrepancies resolved by a third investigator (J. F.).

### Data Extraction

Two review authors (L.H. and Y.G.) independently extracted data from eligible studies. Extracted data included first author, publication year, country, setting of the run-in period, duration of follow-up, dose of patiromer, sample size, participant feature, and outcome variables of interest. The primary outcome was the association between the reduction of hyperkalemia and patiromer comparing with placebo. The secondary outcome was the association between optimization of RAASi therapy (including the incidence of accepting target dose of MRA and proportion of discontinuing of RAASi) and patiromer. The safety outcomes took adverse events (AE), severe adverse events (SAE), AE leading to disconnection, all-cause death, hypokalemia, gastrointestinal disorder, and headache into consideration.

### Risk of Bias and Certainty of Evidence Assessment

The authors (C.L. and Z.Z.) independently performed the quality assessment and risk of bias using the Cochrane Risk of Bias Tool, and disagreements were resolved through the consensus method. Certainty of evidence was evaluated using the Grading of Recommendations Assessment, Development, and Evaluation (GRADE) framework, which divides evidence into very low, low, moderate, and high levels.

### Subgroup Analyses

Subgroup analyses were performed on the following variables: blinding (single-blind or double-blind); run-in period (with or without run-in period); duration of run-in period (≤4 weeks or >4 weeks); duration of follow-up duration (≤ 8 weeks or > 8 weeks); data source (from an RCT subgroup or a specialized RCT); risk of bias (low or high); participant feature (with or without hyperkalemia).

### Data Synthesis and Analyses

The statistical analyses were performed using Review Manager, version 5.3 (Cochrane Collaboration), and Stata, version 16.0 (College Station, Texas, USA). The heterogeneity across studies was quantified using the I^2^ statistic (0–25% low heterogeneity, 25–50% moderate heterogeneity, 50–75% substantial heterogeneity, 75–100% high heterogeneity). Dichotomous data were analyzed using the Mantel–Haenszel method, and the pooled risk ratios (RR) and corresponding 95% confidence intervals (CI) were then calculated. Publication bias was assessed by Egger’s method. We did not create a funnel plot because we included fewer than ten trials.

## Results

### Literature Search and Study Selection

**Fig. 1:**
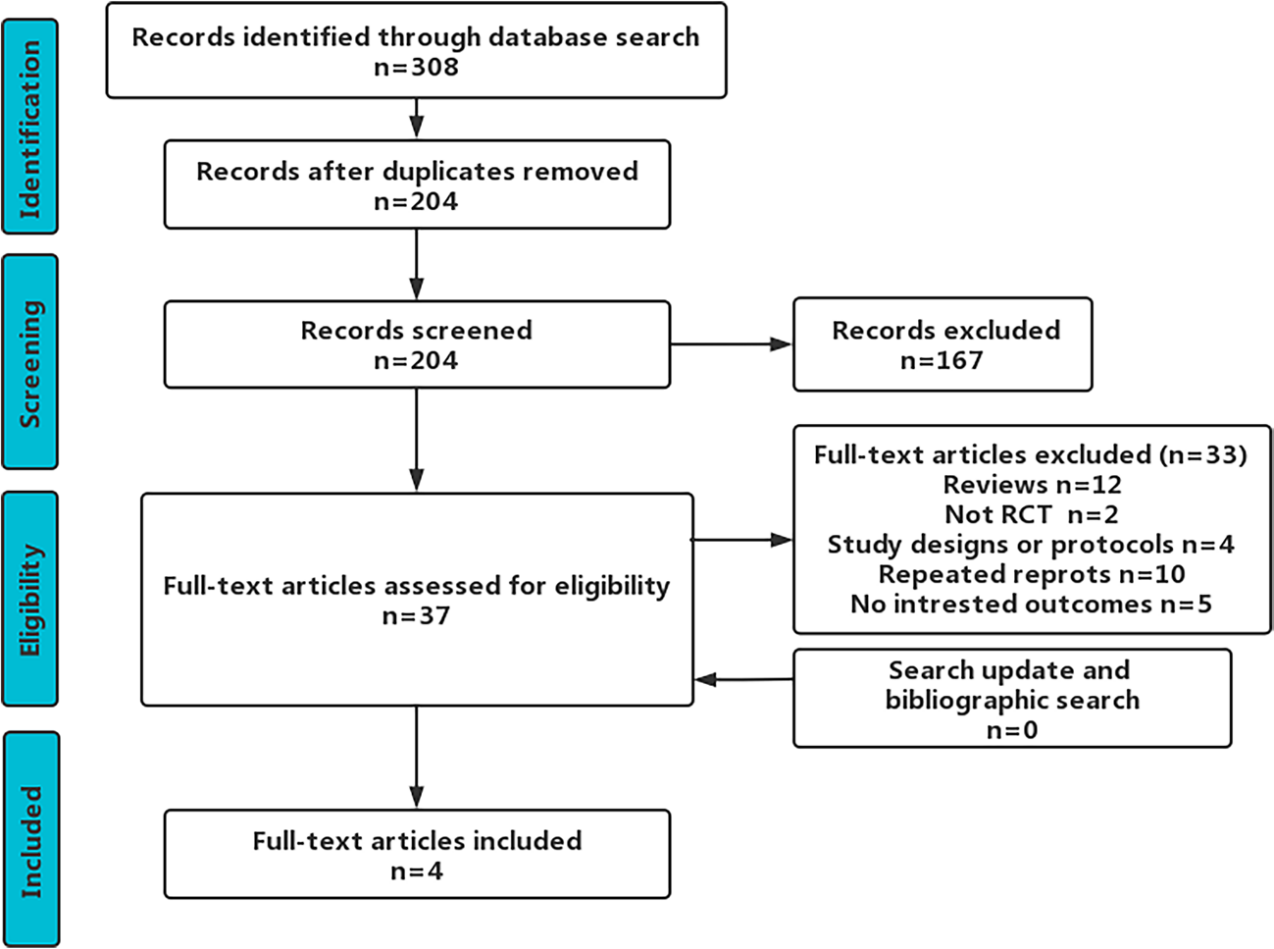
The flow diagram for the study search process

In our initial and update searches, we identified 204 records after removing duplicates. After screening the title and abstract comments, the full text of 37 articles was reviewed. Four studies were eligible for data extraction and quantitative analysis [15-18]. Figure 1 shows the flow of records through the review; Supplementary Appendix S2 includes a list of excluded studies with reasons. Table 1 summarizes the characteristics of the included articles.

**Table 1:** Study characteristics

Reference	Blindness	Study population			Run-in	Follow-up	Intervention	Control	Sample size	Study outcomes
		Heart failure	Serum potassium (mmol/L)	eGFR (mL/min/1.73 m^2^)						
Rossignol et al. 2020 [15]	double-blind	presence	4.3–5.1	25–45	4 weeks	12 weeks	patiromer titrated up to maximum of 25.2g/d	placebo	132	①②④⑤
Butler et al. 2022 [16]	double-blind	NYHA Class II–IV and a LVEF≤ 40%	>5.0 mmol/l or normokalemic at screening but had a history of dose reduction or discontinuation of the RAASi therapy due to hyperkalemia in the previous 12 months	>30	12 weeks	54 weeks	patiromer titrated up to maximum of 25.2g/d	placebo	878	①②③④
Pitt et al. 2015 [17]	single-blind	presence	5.1–6.5	15–60	4 weeks	8 weeks	mild hyperkalemia: patiromer 8.4g/d; moderate-to-sever e hyperkalemia: patiromer 16.8g/d	placebo	49	①②③④
Buysse et al. 2012 [18]	double-blind	NYHA Class II–III and an LVEF of approximately 40%	4.3–5.1	<60	No run-in period	4 weeks	patiromer 30g/d	placebo	105	①②③④

①Reduction of hyperkalemia; ②Optimization of RAASi therapy; ③Mean change in serum potassium; ④Safety outcomes; ⑤LS mean (SE) AOBP reductions

The included studies were compiled from four databases that were published between 2012 and 2022. All of these studies were RCTs, one of which was single-blind, and the others were double-blind. Three of the studies designed the “run-in period” or similar mechanism to screen the population for inclusion, whereby all eligible people were given a certain dose of RAASi and patiromer before entering the placebo-controlled phase; and titrated patiromer and RAASi doses based on serum K^+^. At the end of the run-in period, patients were randomly assigned to patiromer or placebo groups for further study; two studies included patients with normal serum potassium, one study included patients with hyperkalemia, and one study included patients with hyperkalemia or at risk of hyperkalemia.

### Risk of Bias

**Fig. 2:**
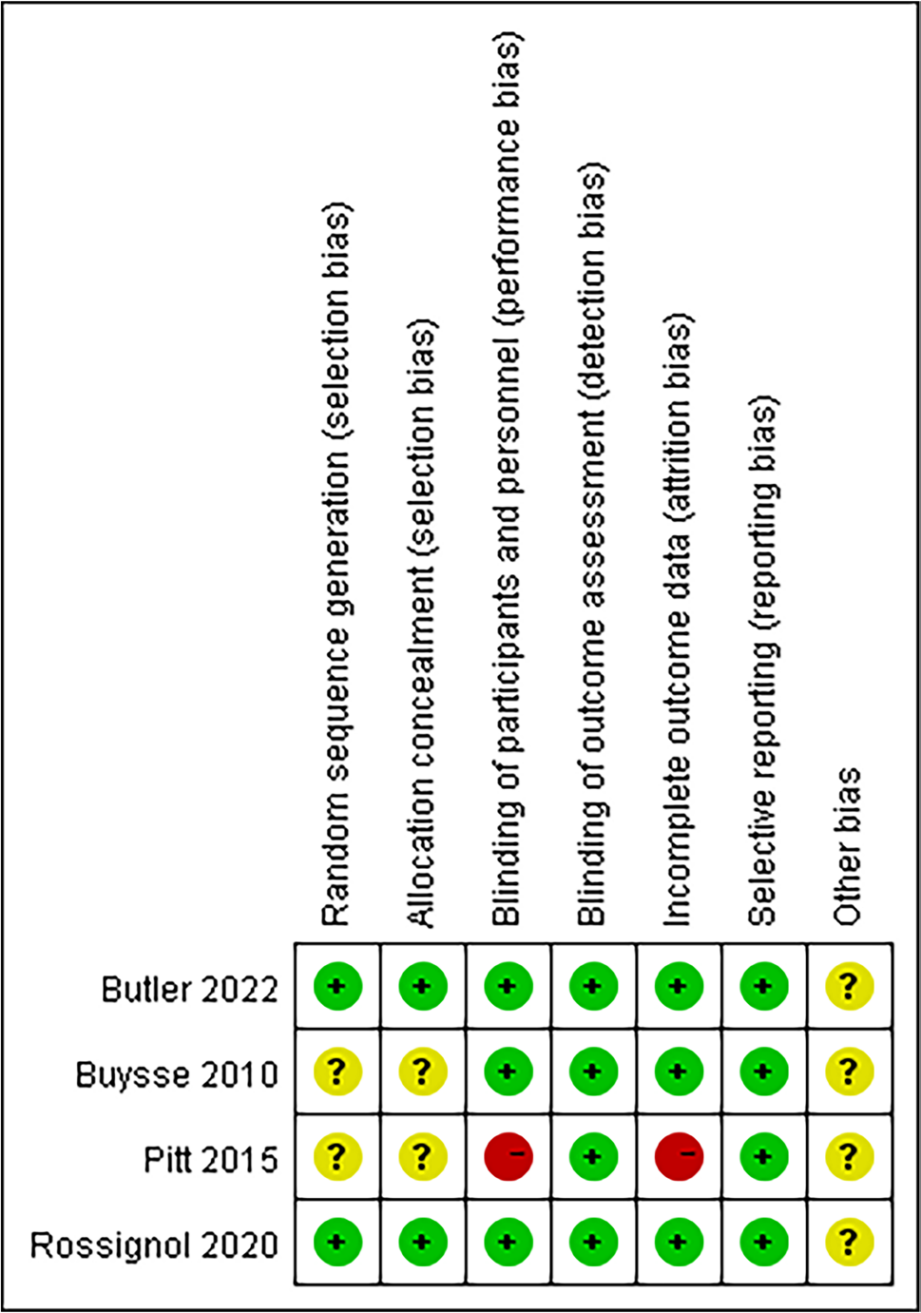
Risk of bias for the included trials

The risk of bias for the included trial is presented in Fig. 2. The description of the randomization process and allocation concealment was presented ambiguously in two RCTs [17, 18]. A single-blind study may have introduced a performance bias [17]. However, since the primary outcome was detected by laboratory methods, the results of this meta-analysis are less likely to be influenced by the single-blind study design. One study whose experimental design may have excluded patients who were insensitive to patiromer was considered to be at high risk of bias [17]. Considering these studies were all sponsored by the pharmaceutical industry, they may contain uncertain risk of bias.

### Primary Outcome

**Fig. 3:**
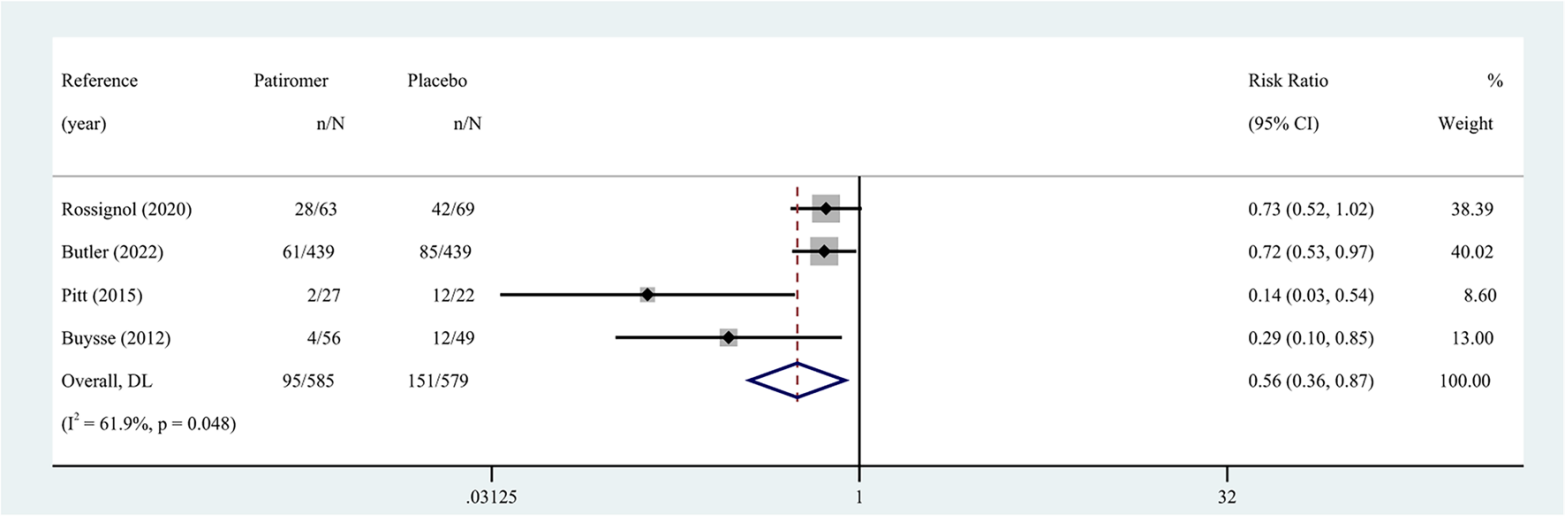
Meta-analysis for the risk of hyperkalemia. The size of the box is proportional to the weight of the study in the meta-analysis. RR, risk ratio; CI, confidence interval

The effect of patiromer on reducing the incidence of hyperkalemia in patients with HF was reported in four eligible studies. Hyperkalemia was defined as serum K^+^ ≥ 5.5 mmol/L. The random-effects model was used to assess the pooled results, which showed a 44% reduction in the overall risk of hyperkalemia in patients (RR 0.56, 95% CI 0.36 to 0.87; I^2^ = 61.9%) (Fig. 3). Pooled results carried substantial heterogeneity. We found evidence of publication bias through Egger’s test (*p* = 0.035 ). In terms of the primary outcome, these findings were considered evidence with moderate proof power. Supplementary Table 6 summarizes the quality of evidence based on the GRADE framework.

### Secondary Outcomes

**Fig. 4:**
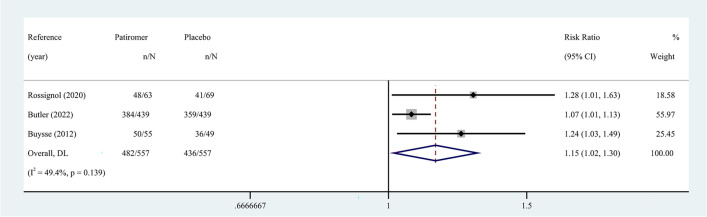
Meta-analysis for tolerance to target doses of MRA

**Fig. 5:**
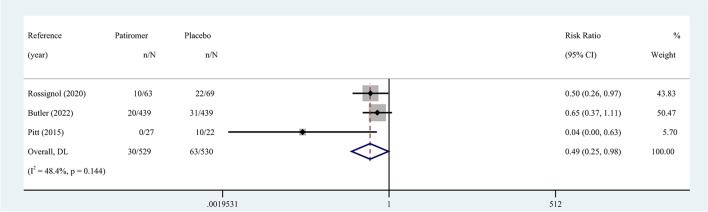
Meta-analysis for incidence of discontinuation of RAASi therapy

Secondary outcomes were assessed by a random-effects model. The target dose of mineralocorticoid receptor antagonist (MRA) was defined as 50 mg of spironolactone or eplerenone. RAASi includes angiotensin-converting enzyme inhibitor (ACEI), angiotensin receptor blocker (ARB), angiotensin receptor-neprilysin inhibitor (ARNI), renin inhibitor, and MRA. Compared with placebo, patients taking patiromer had better tolerance to target doses of MRA (RR 1.15, 95% CI 1.02 to 1.30; I^2^ = 49.4%) (Fig. 4), and the incidence of discontinuation of RAASi therapy decreased by 51% (RR 0.49, 95% CI 0.25 to 0.98; I^2^ = 48.4%) (Fig. 5). Egger’s test showed that the former had no significant publication bias (*P* = 0.077), while the latter had statistically significant publication bias (*P* = 0.031). According to the GRADE framework, the overall quality of the evidence is high and moderate (Supplementary Table 6). In addition, the study by Butler et al. reported patiromer decreased the incidence of MRA decrement (HR 0.62, 95% CI 0.45 to 0.87; *P* = 0.006) [16], while the study by Buysse et al. reported patiromer increased the incidence of spironolactone increment (91% patiromer, 74% placebo; *P* = 0.019) [18]. However, there was no study reporting similar outcomes, and the analysis of pooled results was hindered.

### Safety Outcomes

We examined the incidence of several safety outcomes, including total AE, SAE, AE leading to disconnection, all-cause death, hypokalemia, gastrointestinal disorder, and headache. The entire results for all safety outcomes were shown in Table 2 and Supplementary Fig. 9.

**Table 2:** Meta-analysis for the risk of safety outcomes. Hypokalemia was defined as serum K^+^ ≤ 3.5 mmol/L

Safety outcome	No. of studies (patients)	RR	I^2^	P
Any AE	4 (1136)	1.01 (0.93, 1.09)	51%	0.78
Any SAE	4 (1136)	0.87 (0.63, 1.22)	0	0.43
AE leading to disconnection	4 (1136)	1.24 (0.72, 2.14)	44%	0.45
All-cause death	4 (1136)	1.13 (0.64, 2.02)	0	0.67
Hypokalemia	4 (1136)	1.51 (1.07, 2.12)	0	0.02
Headache	2 (181)	0.37 (0.10, 1.34)	0	0.13
Gastrointestinal disorder	4 (1136)	1.40 (0.95, 2.06)	47%	0.09
Constipation	2 (927)	2.23 (0.82, 6.02)	0	0.11
Nausea	2 (927)	1.38 (0.41, 4.60)	0	0.61
Diarrhoea	3 (1059)	1.21 (0.67, 2.18)	0	0.54

Overall, the patiromer therapy was associated with an increased risk for hypokalemia (RR 1.51, 95% CI 1.07 to 2.12, I^2^ = 0%). There was no evidence demonstrating other significant safety issue differences, such as SAEs, all-cause death or hypomagnesemia, between patiromer therapy and placebo.

Noticeably, data on the incidence of serum potassium ≤ 4.0 mmo/L would be valuable, but only the study by Buysse et al. offered relevant data [18]. Patients taking patiromer were more likely to have serum potassium < 4.0 mmol/L compared to patients in the placebo group (47% patiromer, 10% placebo; *P* < 0.01).

We did not perform the analysis of the pooled results for hypomagnesemia due to the inconsistency in the definition of hypomagnesemia across the included studies. The study by Rossigno et al. and the study by Butler et al. showed no significant difference in the incidence of hypomagnesemia between groups [15, 16]. In the study by Pitt et al., no hypomagnesemia occurred during the follow-up period in both two groups [17], while the study by Buysse et al. showed an obvious difference in the incidence of hypomagnesemia between the two groups(patiromer 24%, placebo 2.1%) [18].

### Sensitivity Analyses

We performed leave-out analyses to explore the sources of heterogeneity. The heterogeneity of the primary outcome was mainly driven by the study by Pitt et al. [17]. If this study were excluded, the I^2^ in the adjusted analysis would be reduced to 25.4% (RR 0.68, 95% CI 0.52 to 0.90; I^2^ = 25.4%). The source of heterogeneity may be related to the design of the trial and participant features.

According to the design of the study by Pitt et al. [17], the patients carrying HF, renal failure, and hyperkalemia at the same time were included. These patients would take a certain dose of patiromer in the run-in period, and if they gained normal serum potassium at the end of the run-in period, they would be eligible to enter the follow-up period, which randomly divided eligible patients into the placebo group or patiromer group. Therefore, patients who were insensitive to patiromer would be screened out after the run-in period, while sensitive patients would be enrolled in the follow-up period. This conjecture is also consistent with the result that the study by Pitt et al. presented the highest RR value [17].

The study by Butler et al. [16] drove the main heterogeneity of the first part of the secondary outcomes, which is the tolerance to target dose MRA, and if this study is excluded, heterogeneity would be reduced from 49.4% to 0% in adjusted analysis (RR 1.25, 95% CI 1.08 to 1.45; I^2^ = 0%). A longer follow-up period may contribute to higher heterogeneity, and in the subsequent subgroup analyses, we adjusted the relevant factors.

After excluding studies one by one, we found that the main source of heterogeneity in the second part of the secondary outcomes, which is the proportion of discontinuing of RAASi, was the study by Pitt et al. [17]. After removing this trial, I^2^ decreased to 0% (RR 0.58, 95% CI 0.38 to 0.89; I^2^ = 0%). By comparing the research characteristics, it was supposed that the heterogeneity is related to the design of the study by Pitt et al. above-mentioned, which screened out patients insensitive to patiromer before entering the randomized controlled period. In addition, the study by Butler [16] and the study by Rossignol [15] reported the rate of patients who discontinued MRA, while the study by Pitt [17] reported the rate of patients who discontinued various types of RAASi, which might also be a source of heterogeneity.

### Subgroup Analyses

To investigate the subgroup differences in the outcomes, we conducted subgroup analyses according to the characteristics of eligible studies, including blinding, run-in period, participant feature and risk of bias.

The results of subgroup analyses of the primary outcome are shown in Table 3 and Supplementary Fig. 6. In subgroup analyses of the primary outcome, there were significant differences between subgroups in blinding (*P* = 0.02), risk of bias (*P* = 0.02), and duration of the follow-up period (*P* = 0.006). Coincidentally, the blinding subgroups contain exactly the same trials as the subgroups of risk of bias. As the primary outcome was detected by laboratory methods, the results of this meta-analysis are unlikely to have been influenced by the single-blind study design.

**Table 3:**
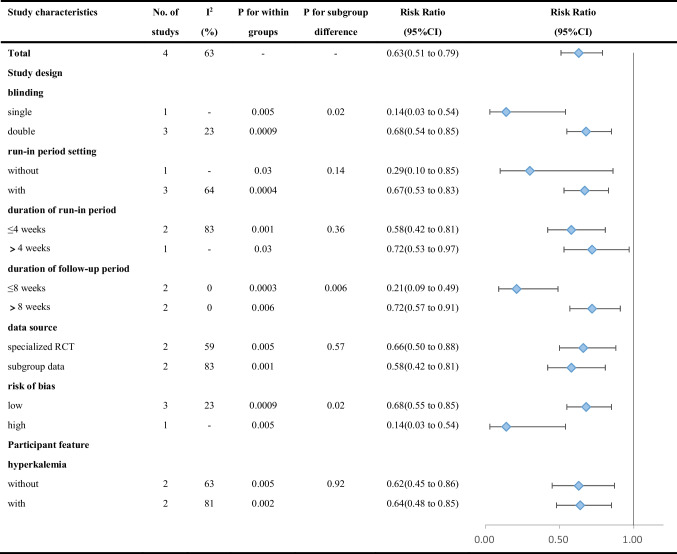
Subgroup analyses of the association between patiromer and incidence of hyperkalemia according to study characteristics

As mentioned in the sensitivity analysis, the study by Pitt et al. [17] might be a major source of heterogeneity due to the study design, which might introduce the risk of bias. Thus, we adjusted the risk of bias in the subgroup analyses, and statistically significant differences between the subgroups were apparent during the follow-up period, while I^2^ decreased to 0% inside each subgroup.

These discoveries revealed a possible trend: there was a difference between the long-term and short-term effects of patiromer in reducing the incidence of hyperkalemia in patients with HF.

The first part of the results of subgroup analyses of the secondary outcomes are presented in Table 4 and Supplementary Fig. 7, while the second part in Table 5 and Supplementary Fig. 8. There was no statistically significant difference between the subgroups for the secondary outcomes.

**Table 4:**
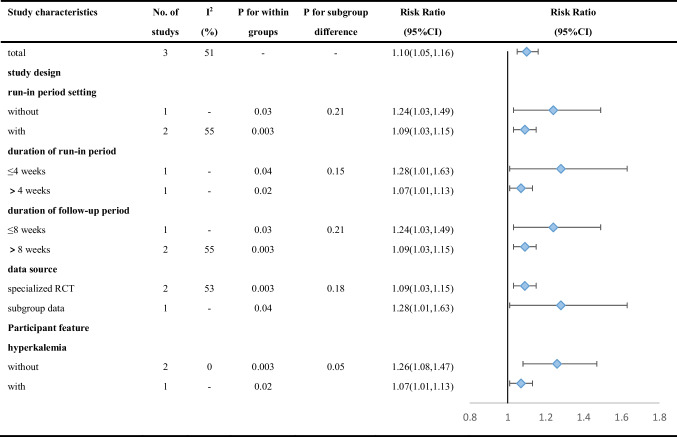
Subgroup analyses of the association between patiromer and tolerance of target dose of MRA according to study characteristics

**Table 5:**
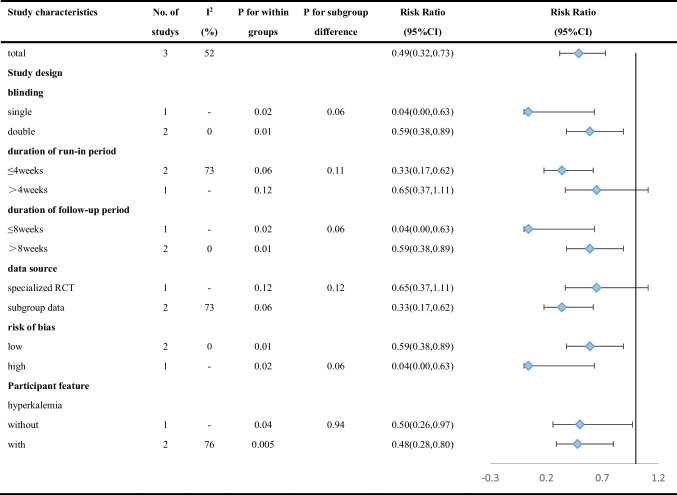
Subgroup analyses of the association between patiromer and incidence of discontinuation of RAASi therapy according to study characteristics

## Discussion

### Principal Findings

In the present meta-analysis of four studies enrolling 1136 patients with HF, patiromer therapy resulted in a potential reduction in the incidence of hyperkalemia. In addition, patiromer therapy was also associated with optimization of RAASi therapy (including increasing the proportion of tolerance of target dose MRA and reducing the ratio of RAASi discontinuation). Compared to placebo, the incidence of hypokalemia was significantly higher under patiromer therapy. Our study demonstrated that the incidence of other AEs under patiromer therapy was generally similar to using placebo.

### Comparison with Other Studies

In recent years, several clinical trials have reported the effects of patiromer in lowering serum potassium, reducing the incidence of hyperkalemia, and optimizing RAASi therapy in patients with HF.

A previous meta-analysis based on three studies concluded that patiromer as a novel potassium binder could optimize RAASi therapy in patients with HF (RR 1.25, 1.08 to 1.45) [19]. However, this study included only three studies, with a lack of heterogeneity, limited follow-up period (1–3 months), and small numbers of events and patients, which we believe, hindered the drawing of valid conclusions [21]. In addition, they performed subgroups of the types of potassium binders only, which we believe may, to some extent, omitted some outcomes with clinical value.

Our study has several advantages over previous meta-analyses. First, we comprehensively and systematically studied the effect of patiromer on reducing the incidence of hyperkalemia and optimizing RAASi therapy in HF patients, obtaining pooled results of higher accuracy by excluding confounding factors such as different types of novel potassium binders and various outcomes. Second, we are confident that our results are reliable because the included studies were all RCTs, only one of which had a high risk of bias; despite the high heterogeneity of the pooled results, the sources of heterogeneity were all reasonably justified in sensitivity analyses and subgroup analyses, and the pooled results remained stable without directional changes after excluding studies that primarily drove heterogeneity.

### Underlying Mechanisms

#### Pathophysiology Mechanisms

The renin-angiotensin system plays a vital role in potassium metabolism in patients with HF. Commonly, according to pathophysiological mechanisms, patients with HF have lower cardiac output compared to normal persons, which results in renal hypoperfusion, which activates the renin-angiotensin system, thereby promoting potassium excretion by stimulating aldosterone synthesis [22]. However, the application of RAASi, including ACEi, ARB, and MRA, inhibits the synthesis or action of aldosterone, resulting in a reduction in potassium excretion. The inhibition would be especially more apparent under the circumstance of combination therapy [23, 24]; high serum potassium can also directly inhibit RAAS [25], resulting in a tendency to further elevate serum potassium.

Accumulating evidence supports the link between disturbances in potassium metabolism and adverse clinical outcomes in patients with HF. Several studies reported a U-shaped association between serum potassium and adverse clinical outcomes in patients with HF [4, 26–28], that is, the incidence of adverse clinical outcomes in patients with HF was relatively low in a narrow range.

It is worth noting that in the observational study by Cooper et al., after covariate adjustment, hyperkalemia was found associated only with the rise of short-term but not long-term mortality [4]. Elucidating this causal relationship is of particular clinical importance because it remains unclear whether treatment targeting hyperkalemia can increase the long-term survival rate of patients with HF.

Although it is uncertain whether hyperkalemia is a risk factor or a sign of increased risk for adverse clinical outcomes in patients with HF, certain mechanisms that may increase such risk should be paid attention to.

Hyperkalemia has a grand effect on cardiac electrophysiology, including a decrease in myocardial resting membrane potential, increased cardiac depolarization, myocardial excitability, cardiac instability, and conduction system abnormalities, which could ultimately lead to arrhythmias, and even progress to ventricular fibrillation and asystole [29, 30].

Even if it is indeterminate whether therapies targeting hyperkalemia themselves could directly improve clinical outcomes, such therapies may make it possible for patients with HF to tolerate higher doses of RAASi, thus providing indirect clinical benefits [24].

#### Drug Mechanisms

Patiromer is a type of non-absorbable, low expansion ratio, cross-linked polymer, composed of beads with a diameter of about 118 µm, with fine fluidity and appropriate viscosity, and is stable in physiological environments.

Its main mechanism is to exchange Ca^2+^ for K^+^ in the digestive tract (mainly in the colon, where the concentration of K^+^ is the highest) and promote the excretion of K^+^ from the feces [14, 24]. Compared with sodium polystyrene sulfonate (SPS), patiromer carries physical properties such as limited water absorption and low expansion ratio; therefore, the digestive tract reactions it may cause are relatively low. Another advantage of patiromer is that its exchange ion is Ca^2+^ instead of Na^+^; thus, it may be more applicable in patients with high volume load, such as HF, severe hypertension and edema. At physiological pH, each gram of patiromer can bind 8.5–8.8 mEq K^+^ in vitro, which is much higher than SPS and other resins [24].

Vitro drug–drug interaction studies showed the binding rate of patiromer with multiple types of drug cannot be ignored [24]. Based on the vitro data, FDA recommended such types of drug should be taken at least 6 h before or after patiromer [24, 31].

It is worth noting that patiromer and SZC have considerable distinctions in pharmacokinetic, pharmacodynamic, and safety profile respects, rendering them dissimilar. Despite that their pharmacologic mechanisms work similarly such as exchanging cations for potassium in the gastrointestinal tract, binding potassium, and increasing its fecal excretion, the cations they exchange potassium for are totally different: patiromer for Ca^2+^, SZC for Na^+^ [23]. As a consequence, the incidence of edema would be much higher in patients under SZC therapy due to increased absorption of Na^+^ [24]. Since HF patients are often volume overloaded, the net clinical benefit of lowering serum potassium with patiromer and SZC might not be equivalent. Min et al. Reported that the initiation of SZC might be associated with an increased risk of hospitalization for heart failure and severe edema compared to patiromer in routine practice in a cohort study [32]. A previous meta-analysis expounded that comparing with standard of care, patiromer had lower rates of hyperkalemia, while no significant differences between the two groups in terms of overall adverse effects, any serious/specific adverse effects, or treatment discontinuation as a result of adverse effects was observed [33]. However, SZC exhibited varying efficacy and safety. Compared with standard care, SZC showed no significant difference in the occurrence of hyperkalemia during treatment, overall adverse effects, any serious/specific adverse effects, or treatment discontinuation as a result of adverse effects, but showed a higher rate of edema in the treatment group. In addition, the incidence of edema in patients treated with SZC was dose-dependent [33].

#### Initiation and Monitoring

Recent studies indicated that the incidence of adverse clinical outcomes in patients with HF was relatively low in a narrow range of serum potassium. Although the optimal range of serum potassium reported by these studies is not completely identical, it is generally relatively safe between 4.0 and 5.0 mmol/L [4, 26, 34]. An expert panel proposed a treatment algorithm for chronic hyperkalemia that builds upon the HF guidelines, expert consensus, and clinical practice [35–38]. For common HF patients with mild hyperkalemia, serum K^+^ 5.1–5.5 mEq/L is considered acceptable due to relatively low associated risk and should not limit RAASi titration. It is considered acceptable, under such circumstance, to conduct a low-potassium diet or regular therapies (such as initiating a diuretic or increasing its dose) to adjust serum potassium. However, under two circumstances, the algorithm advocated the use of patiromer to enable RAASi titration, which are, first, for heart failure, concomitant chronic kidney disease (CKD) stage 3b–4, and/or diabetes mellitus (DM) patients with mild hyperkalemia and second, serum K^+^ levels > 5.5 mmol/L (moderate and severe hyperkalemia). In patients experiencing severe hyperkalemia (serum K^+^ > 6.0 mEq/L), RAASi therapy should be discontinued or reduced, and patiromer initiated. Following normalization of serum K^+^ levels, crucial RAASi therapy should be re-initiated and titrated to maximal doses with close monitoring of serum K^+^ levels.

Low serum potassium is a side effect that is reversible and readily managed in HF patients by reducing the patiromer dosage or up-titrated RAASi therapy [16, 39]. Clinicians have the discretion to adjust the dose of patiromer from 8.4 g to 25.2 g as needed to maintain potassium within a safe range of 4.0 to 5.0 mmol/L [40].

Serum potassium and creatinine monitoring would help reduce both unwanted extremities of serum potassium. Although current guideline for the exact timing of monitoring serum potassium and renal function after initiating patiromer remains insufficient, the following suggestions could be offered referring to expert recommendations and guidelines for RAASi titration [35, 39–43]: if the potassium level is very elevated above 6.0 mmol/L or other clinically worrisome factors are present, a repeat potassium level check should be drawn within 12 h to monitor efficacy. In the outpatient setting, following initiation or adjustment of patiromer therapy or RAASi therapy, performing serum potassium and creatinine monitoring at 48 to 72 h, and repeating after 1 week, 1 month, and every 3 to 4 months thereafter would be reasonable.

#### Limitations of this Study

We acknowledge the presence of limitations in our study. First, long-term conclusions could not be drawn due to the limited duration of the follow-up periods in three of the trial. In this regard, the duration of the follow-up period of the study by Butler et al. was several times longer than that of other studies, and the duration of follow-up period subgroups of the primary outcome reflected statistically significant differences.

Second, because few RCTs provided HF indicators, including LVEF, BNP, etc., before and after patiromer therapy, the consolidation of relevant data was limited, which made it difficult to assess the benefits of patiromer in reducing potassium and optimizing RAASi therapy.

Third, some subgroup analyses with clinical value, such as the evaluation of patiromer efficacy in HF patients with or without chronic kidney disease were hindered due to limitations of the number of included studies and participant features.

Fourth, because the population included in the RCTs with chronic kidney disease was of non-negligible proportion, more caution is needed in generalizing the results of this study to the entire population of HF patients.

However, despite that the study designs, data materials, follow-up periods, and study qualities varied and the limitations that existed, the relatively stable study results indicate that our findings were statistically reliable.

Therefore, we need additional data from postmarketing surveillance to assess the long-term effects of patiromer and the incidence of rare AEs. We expect the quality of the evidence to be improved with future updates and more high-quality studies.

## Conclusion

Our systematic review and meta-analysis showed that patiromer has considerable effects on reducing the incidence of hyperkalemia and optimizing RAASi therapy in patients with HF. Subgroup analyses indicated that comparing the long-term and short-term effects of patiromer, in terms of reducing the incidence of hyperkalemia in the HF population, the two present differently; but on the outcome of optimizing RAASi therapy, there was no significant difference.

For the HF patients receiving patiromer therapy, the only AE, presently observed, of statistically significant differences, compared with placebo, was hypokalemia, since there is currently no evidence that other AEs of statistically significant differences exist. Regular monitoring of serum K^+^ in patients taking patiromer is necessary, considering that hypokalemia is an independent risk factor for adverse clinical events in patients with heart failure [4].

Sufficient RCTs are needed in the future to assess the long-term effects and potential harms of patiromer to improve clinical outcomes in HF patients with, or at risk of hyperkalemia.

## Supplementary Information

ESM 1 (DOC 14874 kb)

## References

[1] Ziaeian B, Fonarow GC. Epidemiology and aetiology of heart failure. Nat Rev Cardiol. 2016;13(6):368–78. 26935038 10.1038/nrcardio.2016.25 PMC4868779

[2] Rakisheva A, Marketou M, Klimenko A, Troyanova-Shchutskaia T, Vardas P. Hyperkalemia in heart failure: foe or friend? Clin Cardiol. 2020;43(7):666–75. 32445223 10.1002/clc.23392 PMC7368299

[3] Savarese G, Xu H, Trevisan M, et al. Incidence, predictors, and outcome associations of dyskalemia in heart failure with preserved, mid-range, and reduced ejection fraction. JACC Heart Fail. 2019;7(1):65–76. 30553905 10.1016/j.jchf.2018.10.003

[4] Cooper LB, Benson L, Mentz RJ, et al. Association between potassium level and outcomes in heart failure with reduced ejection fraction: a cohort study from the Swedish Heart Failure Registry. Eur J Heart Fail. 2020;22(8):1390–8. 32078214 10.1002/ejhf.1757

[5] McMurray JJ, Packer M, Desai AS, et al. Angiotensin-neprilysin inhibition versus enalapril in heart failure. N Engl J Med. 2014;371(11):993–1004. 25176015 10.1056/NEJMoa1409077

[6] Ferreira JP, Mogensen UM, Jhund PS, et al. Serum potassium in the PARADIGM-HF trial. Eur J Heart Fail. 2020;22(11):2056–64. 32809261 10.1002/ejhf.1987 PMC7756204

[7] Lund LH, Donal E, Oger E, et al. Association between cardiovascular vs. non-cardiovascular co-morbidities and outcomes in heart failure with preserved ejection fraction. Eur J Heart Fail. 2014;16(9):992–1001. 25046483 10.1002/ejhf.137

[8] Desai AS, Liu J, Pfeffer MA, et al. Incident hyperkalemia, hypokalemia, and clinical outcomes during spironolactone treatment of heart failure with preserved ejection fraction: analysis of the TOPCAT trial. J Card Fail. 2018;24(5):313–20. 29572190 10.1016/j.cardfail.2018.03.002

[9] Pitt B, Pfeffer MA, Assmann SF, et al. Spironolactone for heart failure with preserved ejection fraction. N Engl J Med. 2014;370(15):1383–92. 24716680 10.1056/NEJMoa1313731

[10] 2022 AHA/ACC/HFSA guideline for the management of heart failure. J Card Fail. 2022;28(5):e1-e167. 10.1016/j.cardfail.2022.02.010 35378257

[11] Ouwerkerk W, Voors AA, Anker SD, et al. Determinants and clinical outcome of uptitration of ACE-inhibitors and beta-blockers in patients with heart failure: a prospective European study. Eur Heart J. 2017;38(24):1883–90. 28329163 10.1093/eurheartj/ehx026

[12] Eschalier R, McMurray JJ, Swedberg K, et al. Safety and efficacy of eplerenone in patients at high risk for hyperkalemia and/or worsening renal function: analyses of the EMPHASIS-HF study subgroups (Eplerenone in Mild Patients Hospitalization And SurvIval Study in Heart Failure). J Am Coll Cardiol. 2013;62(17):1585–93. 23810881 10.1016/j.jacc.2013.04.086

[13] Beusekamp JC, Tromp J, van der Wal HH, et al. Potassium and the use of renin-angiotensin-aldosterone system inhibitors in heart failure with reduced ejection fraction: data from BIOSTAT-CHF. Eur J Heart Fail. 2018;20(5):923–30. 29327797 10.1002/ejhf.1079

[14] Li L, Harrison SD, Cope MJ, et al. Mechanism of action and pharmacology of patiromer, a nonabsorbed cross-linked polymer that lowers serum potassium concentration in patients with hyperkalemia. J Cardiovasc Pharmacol Ther. 2016;21(5):456–65. 26856345 10.1177/1074248416629549 PMC4976659

[15] Rossignol P, Williams B, Mayo MR, et al. Patiromer versus placebo to enable spironolactone use in patients with resistant hypertension and chronic kidney disease (AMBER): results in the pre-specified subgroup with heart failure. Eur J Heart Fail. 2020;22(8):1462–71. 32452085 10.1002/ejhf.1860 PMC7540031

[16] Butler J, Anker SD, Lund LH, et al. Patiromer for the management of hyperkalemia in heart failure with reduced ejection fraction: the DIAMOND trial. Eur Heart J. 2022;43(41):4362–73. 35900838 10.1093/eurheartj/ehac401 PMC9622299

[17] Pitt B, Bakris GL, Bushinsky DA, et al. Effect of patiromer on reducing serum potassium and preventing recurrent hyperkalaemia in patients with heart failure and chronic kidney disease on RAAS inhibitors. Eur J Heart Fail. 2015;17(10):1057–65. 26459796 10.1002/ejhf.402 PMC5057342

[18] Buysse JM, Huang IZ, Pitt B. PEARL-HF: prevention of hyperkalemia in patients with heart failure using a novel polymeric potassium binder, RLY5016. Future Cardiol. 2012;8(1):17–28. 22185443 10.2217/fca.11.71

[19] Montagnani A, Frasson S, Gussoni G, Manfellotto D. Optimization of RAASi therapy with new potassium binders for patients with heart failure and hyperkalemia: rapid review and meta-analysis. J Clin Med. 2021;10(23). 10.3390/jcm10235483 PMC8658658 34884184

[20] Moher D, Liberati A, Tetzlaff J, Altman DG. Preferred reporting items for systematic reviews and meta-analyses: the PRISMA statement. J Clin Epidemiol. 2009;62(10):1006–12. 19631508 10.1016/j.jclinepi.2009.06.005

[21] Zarzuela D, Chin A. Comment on Montagnani et al. Optimization of RAASi therapy with new potassium binders for patients with heart failure and hyperkalemia: rapid review and meta-analysis. J Clin Med. 2021, 10:5483. J Clin Med. 2022;11(10). 10.3390/jcm11102755 PMC9145906 35628883

[22] Dargie HJ. Interrelation of electrolytes and renin-angiotensin system in congestive heart failure. Am J Cardiol. 1990;65(10):28E–32E. discussion 52E. 2178375 10.1016/0002-9149(90)90249-z

[23] Zannad F, Ferreira JP, Pitt B. Potassium binders for the prevention of hyperkalaemia in heart failure patients: implementation issues and future developments. Eur Heart J Suppl. 2019;21(Suppl A):A55–a60. 30837806 10.1093/eurheartj/suy034 PMC6392413

[24] Sfairopoulos D, Arseniou A, Korantzopoulos P. Serum potassium and heart failure: association, causation, and clinical implications. Heart Fail Rev. 2021;26(3):479–86. 33098029 10.1007/s10741-020-10039-9

[25] Young DB, Smith MJ Jr, Jackson TE, Scott RE. Multiplicative interaction between angiotensin II and K concentration in stimulation of aldosterone. Am J Physiol. 1984;247(3 Pt 1):E328–35. 6476112 10.1152/ajpendo.1984.247.3.E328

[26] Aldahl M, Jensen AC, Davidsen L, et al. Associations of serum potassium levels with mortality in chronic heart failure patients. Eur Heart J. 2017;38(38):2890–6. 29019614 10.1093/eurheartj/ehx460

[27] Krogager ML, Eggers-Kaas L, Aasbjerg K, et al. Short-term mortality risk of serum potassium levels in acute heart failure following myocardial infarction. Eur Heart J Cardiovasc Pharmacother. 2015;1(4):245–51. 27418967 10.1093/ehjcvp/pvv026 PMC4900739

[28] Linde C, Qin L, Bakhai A, et al. Serum potassium and clinical outcomes in heart failure patients: results of risk calculations in 21 334 patients in the UK. ESC Heart Fail. 2019;6(2):280–90. 30629342 10.1002/ehf2.12402 PMC6437434

[29] Dittrich KL, Walls RM. Hyperkalemia: ECG manifestations and clinical considerations. J Emerg Med. 1986;4(6):449–55. 3559133 10.1016/0736-4679(86)90174-5

[30] Widimský J, Cífková R. The heart in hypertension and arrhythmias. Herz. 1990;15(1):49–53. 2138116

[31] FDA. Veltassa (patiromer for oral suspension). Clinical pharmacology and biopharmaceutics review approved by FDA Center for Drug Evaluation and Research (CDER). 2015. Available at: http://www.accessdata.fda.gov/drugsatfda_docs/nda/2015/205739Orig1s000ClinPharmR.pdf. Accessed March 25, 2023.

[32] Zhuo M, Kim SC, Patorno E, Paik JM. Risk of hospitalization for heart failure in patients with hyperkalemia treated with sodium zirconium cyclosilicate versus patiromer. J Card Fail. 2022;28(9):1414–23. 35470055 10.1016/j.cardfail.2022.04.003

[33] Shrestha DB, Budhathoki P, Sedhai YR, et al. Patiromer and sodium zirconium cyclosilicate in treatment of hyperkalemia: a systematic review and meta-analysis. Curr Ther Res Clin Exp. 2021;95:100635. 34367383 10.1016/j.curtheres.2021.100635 PMC8326359

[34] Collins AJ, Pitt B, Reaven N, et al. Association of serum potassium with all-cause mortality in patients with and without heart failure, chronic kidney disease, and/or diabetes. Am J Nephrol. 2017;46(3):213–21. 28866674 10.1159/000479802 PMC5637309

[35] Silva-Cardoso J, Brito D, Frazão JM, et al. Management of RAASi-associated hyperkalemia in patients with cardiovascular disease. Heart Fail Rev. 2021;26(4):891–6. 33599908 10.1007/s10741-020-10069-3 PMC8149346

[36] Ponikowski P, Voors AA, Anker SD, et al. 2016 ESC Guidelines for the diagnosis and treatment of acute and chronic heart failure: The Task Force for the diagnosis and treatment of acute and chronic heart failure of the European Society of Cardiology (ESC) Developed with the special contribution of the Heart Failure Association (HFA) of the ESC. Eur Heart J. 2016;37(27):2129–200. 27206819 10.1093/eurheartj/ehw128

[37] Seferovic PM, Ponikowski P, Anker SD, et al. Clinical practice update on heart failure 2019: pharmacotherapy, procedures, devices and patient management. An expert consensus meeting report of the Heart Failure Association of the European Society of Cardiology. Eur J Heart Fail. 2019;21(10):1169–86. 31129923 10.1002/ejhf.1531

[38] Rosano GMC, Tamargo J, Kjeldsen KP, et al. Expert consensus document on the management of hyperkalaemia in patients with cardiovascular disease treated with renin angiotensin aldosterone system inhibitors: coordinated by the Working Group on Cardiovascular Pharmacotherapy of the European Society of Cardiology. Eur Heart J Cardiovasc Pharmacother. 2018;4(3):180–8. 29726985 10.1093/ehjcvp/pvy015 PMC13588647

[39] Ferreira JP, Butler J, Rossignol P, et al. Abnormalities of potassium in heart failure: JACC State-of-the-Art Review. J Am Coll Cardiol. 2020;75(22):2836–50. 32498812 10.1016/j.jacc.2020.04.021

[40] Colbert GB, Patel D, Lerma EV. Patiromer for the treatment of hyperkalemia. Expert Rev Clin Pharmacol. 2020;13(6):563–70. 32511052 10.1080/17512433.2020.1774363

[41] Yancy CW, Jessup M, Bozkurt B, et al. 2013 ACCF/AHA guideline for the management of heart failure: executive summary: a report of the American College of Cardiology Foundation/American Heart Association Task Force on Practice Guidelines. J Am College Cardiol. 2013;62(16):1495–539. 10.1016/j.jacc.2013.05.019 23747642

[42] Aldahl M, Jensen A-SC, Davidsen L, et al. Associations of serum potassium levels with mortality in chronic heart failure patients. Eur Heart J. 2017;38(38):2890–6. 29019614 10.1093/eurheartj/ehx460

[43] Palmer BF, Carrero JJ, Clegg DJ, et al. Clinical Management of Hyperkalemia. Mayo Clin Proc. 2021;96(3):744–62. 33160639 10.1016/j.mayocp.2020.06.014

