# Supplementary material for: The Efficacy and Safety of Patiromer for Heart Failure Patients: A Systematic Review and Meta-Analysis
Source: Cardiovasc Drugs Ther. 2023 Jun 7;38(6):1245–57. doi: 10.1007/s10557-023-07473-w (PMC11680614; doi:10.1007/s10557-023-07473-w)
Supplement: Supplementary file 1 — (DOC 14874 kb) [file 10557_2023_7473_MOESM1_ESM.doc]

**The Efficacy and Safety of Patiromer for Heart Failure Patients：**

**A Systematic Review and Meta-analysis**

Yuhui Wang^1,2#^ · Yu Gao^1^·Jun Feng^1,2*^· Linlin Hou^1,2^· Chunmiao Luo^1,2^· Zhipeng Zhang^1,2^

^1^ Hefei Hospital Affiliated to Anhui Medical University, Hefei, P.R. China

^2^ The Fifth Clinical College of Anhui Medical University, Hefei, P.R. China

^#^Yuhui Wang should be regard as first author

^*^Corresponding Author: Jun Feng ^1,2^

Tel: +86 13721040287 E-mail: [fengjun0071@sina.com](mailto:fengjun0071@sina.com)

**Supplementary File**

**Figure 6.** Subgroup analyses of association between patiromer and incidence of hyperkalemia according to study characteristics.

**Figure 7.** Subgroup analyses of association between patiromer and tolerance of target dose of MRA according to study characteristics.

**Figure 8.** Subgroup analyses of association between patiromer and incidence of discontinuation of RAASi therapy according to study characteristics.

**Figure 9.** Meta analyses of safety outcomes.

**Appendix S1.** Search strategy.

**Appendix S2.** List of excluded studies with reasons.

**Table 6.** Quality of evidence based on the GRADE framework.


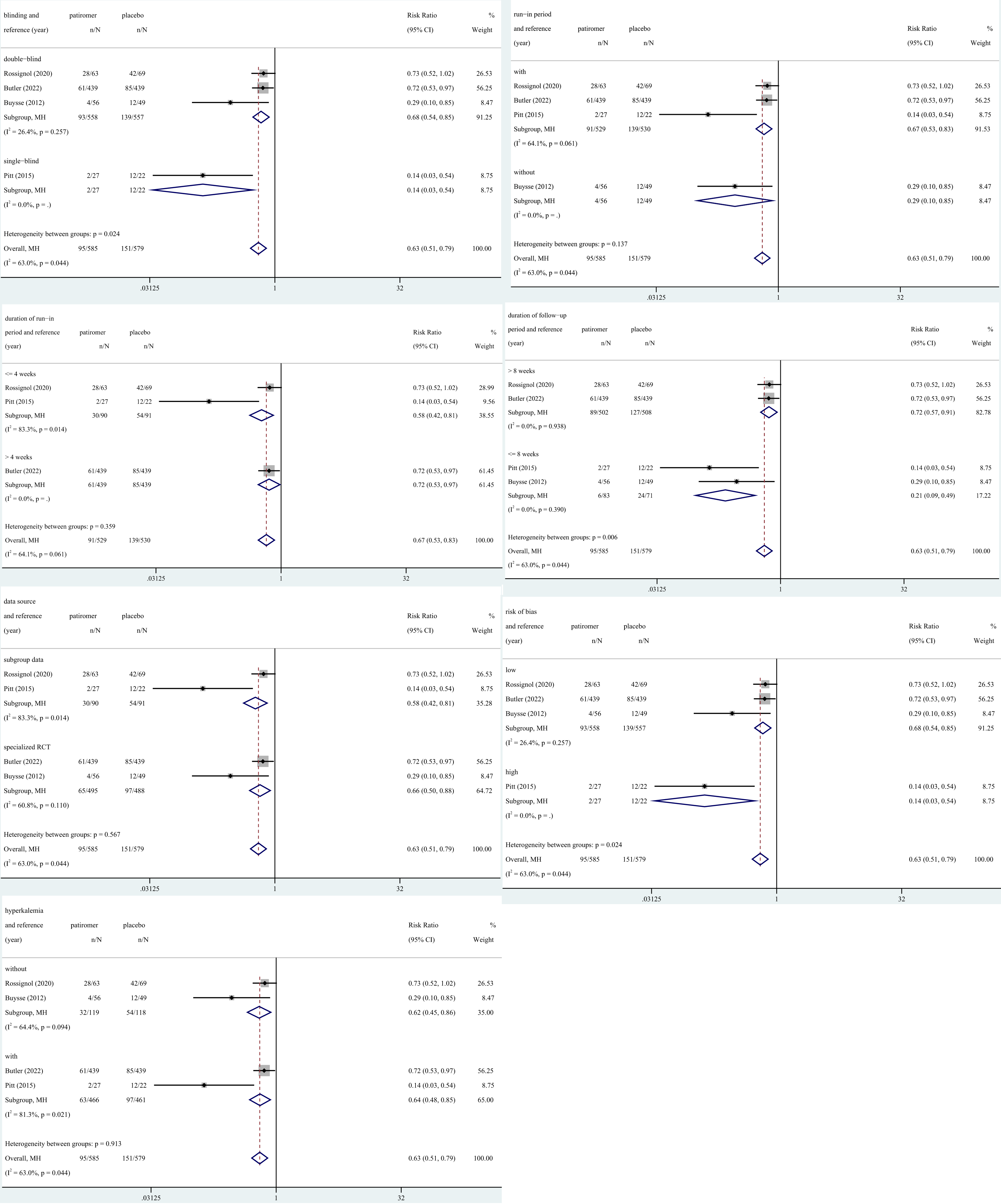


**Figure 6.** Subgroup analyses of association between patiromer and incidence of hyperkalemia according to study characteristics.


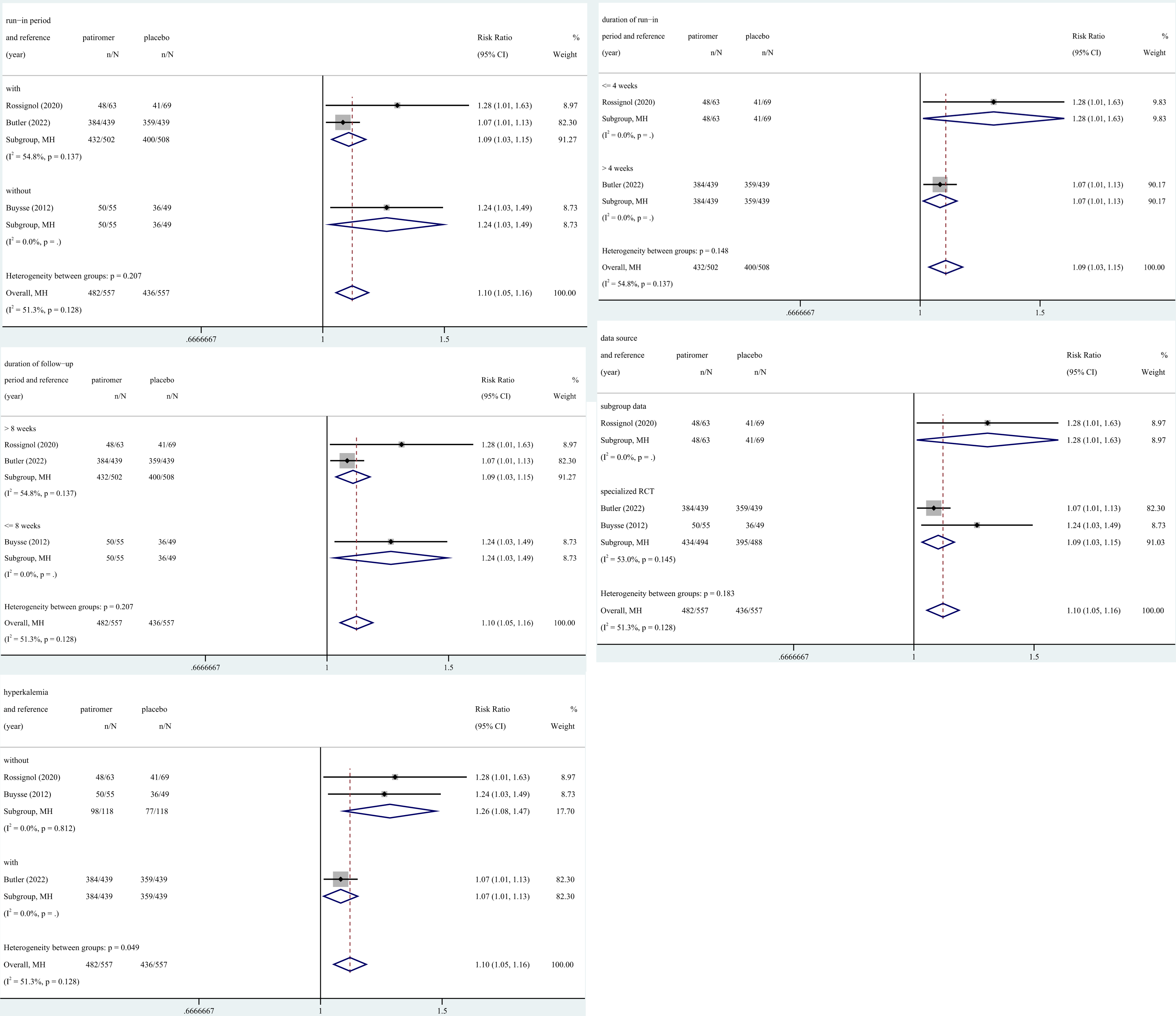


**Figure 7.** Subgroup analyses of association between patiromer and tolerance of target dose of MRA according to study characteristics.


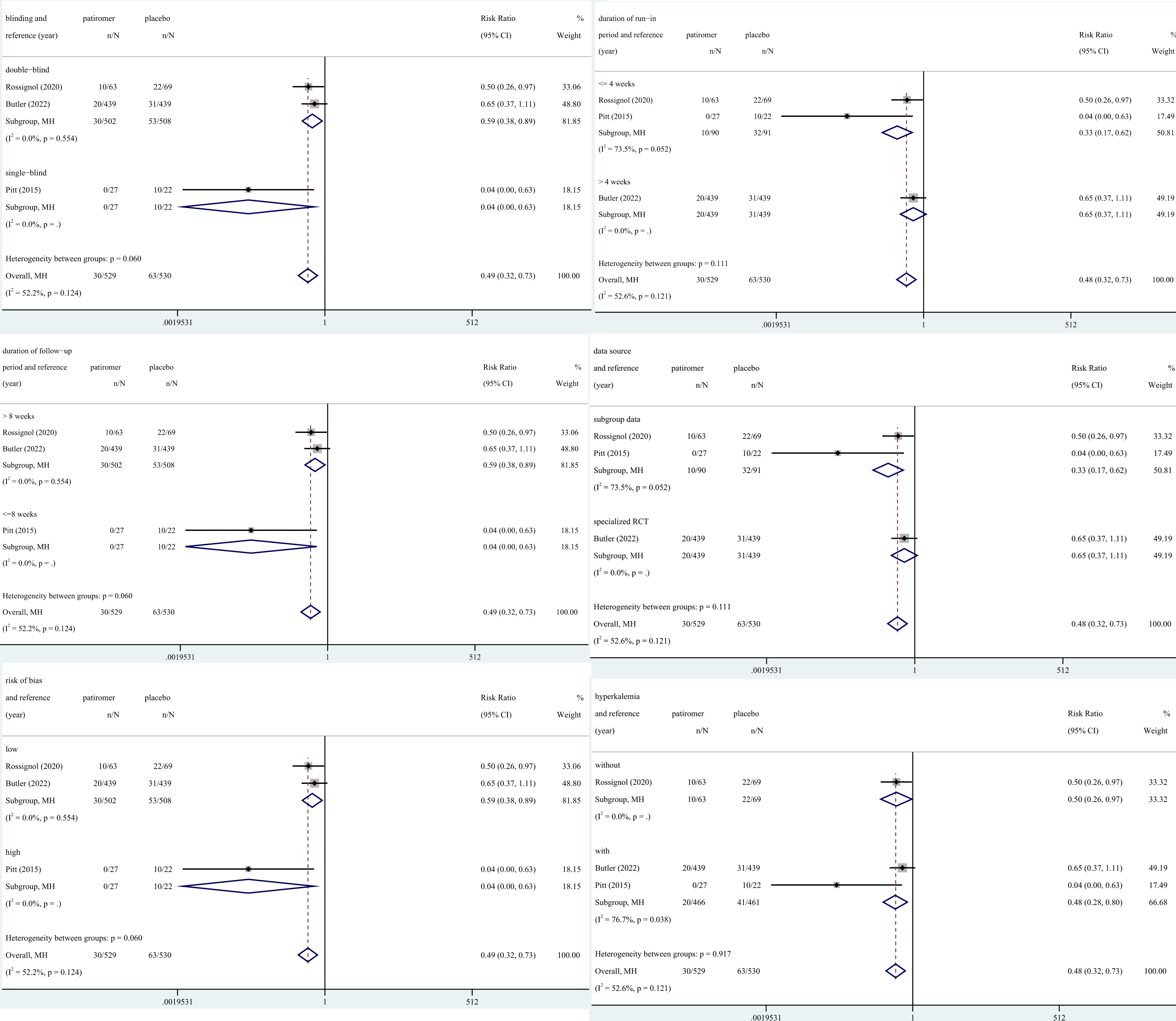


**Figure 8.** Subgroup analyses of association between patiromer and incidence of discontinuation of RAASi therapy according to study characteristics.


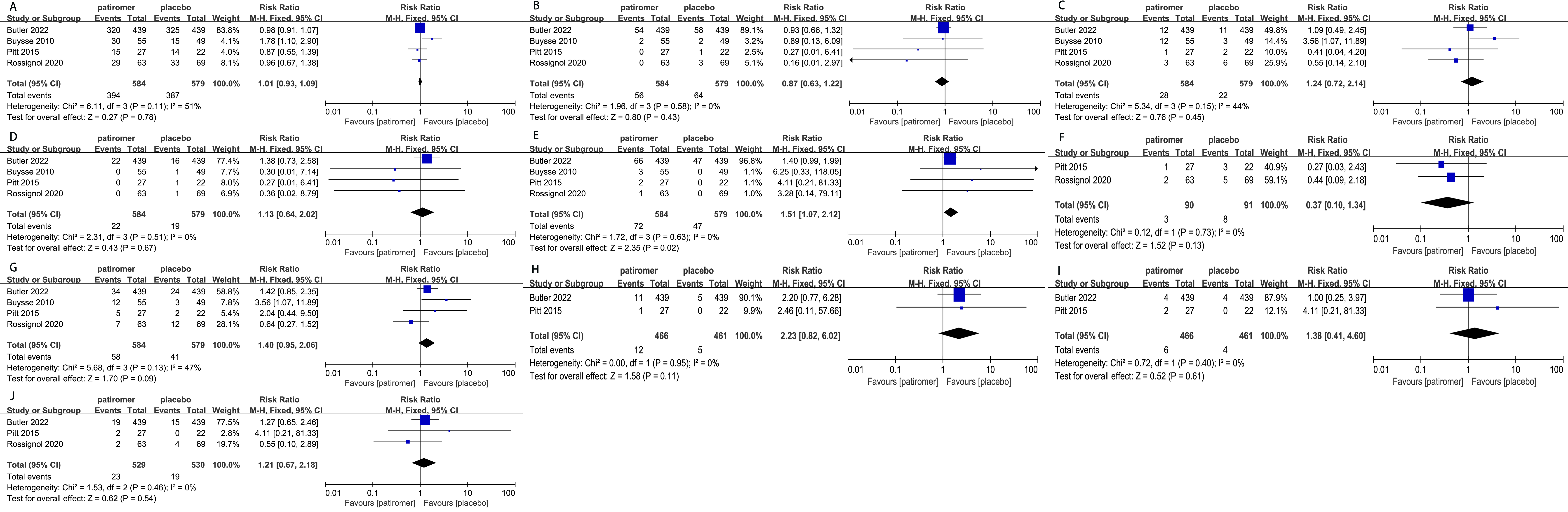


**Figure 9.** Meta analyses of safety outcomes.**A** Any AEs; **B** Any SAEs; **C** AE leading to disconnection; **D** All-cause death; **E** Hypokalemia; **F** Headache; **G** Gastrointestinal disorder; **H** Constipation;**I** Nausea; **J** Diarrhoea.

**Appendix S1.** Search strategy.

Pubmed 2023-3-25 89

(((((((((((((((((((heart failure[MeSH])) OR (heart failure[Title/Abstract])) OR (backward failure, heart[Title/Abstract])) OR (cardiac backward failure[Title/Abstract])) OR (cardiac decompensation[Title/Abstract])) OR (cardiac failure[Title/Abstract])) OR (cardiac incompetence[Title/Abstract])) OR (cardiac insufficiency[Title/Abstract])) OR (cardiac stand still[Title/Abstract])) OR (decompensatio cordis[Title/Abstract])) OR (decompensation, heart[Title/Abstract])) OR (heart backward failure[Title/Abstract])) OR (heart decompensation[Title/Abstract])) OR (heart incompetence[Title/Abstract])) OR (heart insufficiency[Title/Abstract])) OR (insufficientia cardis[Title/Abstract])) OR (myocardial failure[Title/Abstract])) OR (myocardial insufficiency[Title/Abstract])) AND (((((((((((patiromer[MeSH])) OR (patiromer[Title/Abstract])) OR (patiromer acetate[Title/Abstract])) OR (patiromer calcium[Title/Abstract])) OR (patiromer sorbitex calcium[Title/Abstract])) OR (rly 5016[Title/Abstract])) OR (rly 5016s[Title/Abstract])) OR (rly5016[Title/Abstract])) OR (rly5016s[Title/Abstract])) OR (veltassa[Title/Abstract])) AND  ((randomized controlled trial[pt] OR controlled clinical trial[pt] OR randomized[tiab] OR randomised[tiab] OR placebo[tiab] OR drug therapy[sh] OR randomly[tiab] OR trial[tiab] OR groups[tiab]) NOT (animals[mh] NOT humans[mh]))

Web of science 2023-3-25 93

#1 TS=( heart failure OR cardiac backward failure OR cardiac decompensation OR cardiac failure OR cardiac incompetence OR cardiac insufficiency OR cardiac stand still OR decompensatio cordis OR heart backward failure OR heart decompensation OR heart incompetence OR heart insufficiency OR insufficientia cardis OR myocardial failure OR myocardial insufficiency OR backward failure, heart OR decompensation, heart )

#2 TS=( patiromer OR patiromer acetate OR patiromer sorbitex calcium OR rly 5016 OR rly 5016s OR rly5016 OR rly5016s OR veltassa )

#3 TS=(randomised OR randomized OR randomisation OR randomisation OR placebo* OR (random* AND (allocat* OR assign*) ) OR (blind* AND (single OR double OR treble OR triple) ))

#4 #1 AND #2 AND #3

Embase 2023-3-25 75

#1 'heart failure':ti,ab,kw OR 'cardiac backward failure':ti,ab,kw OR 'cardiac decompensation':ti,ab,kw OR 'cardiac failure':ti,ab,kw OR 'cardiac incompetence':ti,ab,kw OR 'cardiac insufficiency':ti,ab,kw OR 'cardiac stand still':ti,ab,kw OR 'decompensatio cordis':ti,ab,kw OR 'heart backward failure':ti,ab,kw OR 'heart decompensation':ti,ab,kw OR 'heart incompetence':ti,ab,kw OR 'heart insufficiency':ti,ab,kw OR 'insufficientia cardis':ti,ab,kw OR 'myocardial failure':ti,ab,kw OR 'myocardial insufficiency':ti,ab,kw OR 'backward failure, heart':ti,ab,kw OR 'decompensation, heart':ti,ab,kw

#2 patiromer:ti,ab,kw OR 'patiromer acetate':ti,ab,kw OR 'patiromer sorbitex calcium':ti,ab,kw OR 'rly 5016':ti,ab,kw OR 'rly 5016s':ti,ab,kw OR rly5016s:ti,ab,kw OR veltassa:ti,ab,kw

#3 'crossover procedure':de OR 'double-blind procedure':de OR 'randomized controlled trial':de OR 'single-blind procedure':de OR random*:de,ab,ti OR factorial*:de,ab,ti OR crossover*:de,ab,ti OR ((cross NEXT/1 over*):de,ab,ti) OR placebo*:de,ab,ti OR ((doubl* NEAR/1 blind*):de,ab,ti) OR ((singl* NEAR/1 blind*):de,ab,ti) OR assign*:de,ab,ti OR allocat*:de,ab,ti OR volunteer*:de,ab,ti

#4 #1 AND #2 AND #3

Cochrane 2023-3-25 52

#1 (heart failure):ti,ab,kw OR (backward failure, heart):ti,ab,kw OR (cardiac backward failure):ti,ab,kw OR (cardiac decompensation):ti,ab,kw OR (cardiac failure):ti,ab,kw (Word variations have been searched) OR (cardiac incompetence):ti,ab,kw OR (cardiac insufficiency):ti,ab,kw OR (cardiac stand still):ti,ab,kw OR (decompensatio cordis):ti,ab,kw OR (decompensation, heart):ti,ab,kw (Word variations have been searched) OR (heart backward failure):ti,ab,kw OR (heart decompensation):ti,ab,kw OR (heart incompetence):ti,ab,kw OR (heart insufficiency):ti,ab,kw OR (insufficientia cardis):ti,ab,kw (Word variations have been searched) OR (myocardial failure):ti,ab,kw OR (myocardial insufficiency):ti,ab,kw (Word variations have been searched)

#2 (patiromer):ti,ab,kw OR (patiromer acetate):ti,ab,kw OR (patiromer calcium):ti,ab,kw OR (patiromer sorbitex calcium):ti,ab,kw OR (rly 5016):ti,ab,kw (Word variations have been searched) OR (rly 5016s):ti,ab,kw OR (rly5016):ti,ab,kw OR (rly5016s):ti,ab,kw OR (veltassa):ti,ab,kw

#3 #1 AND #2

**Appendix S2** List of excluded studies with reasons.

|  | First author | Year | Title | Reason for excluded |
| --- | --- | --- | --- | --- |
| 1 | Agarwal, R. | 2021 | Patiromer and Spironolactone in Resistant Hypertension and Advanced CKD: analysis of the Randomized AMBER Trial | Repeated trail |
| 2 | Agarwal, R. | 2019 | Patiromer versus placebo to enable spironolactone use in patients with resistant hypertension and chronic kidney disease (AMBER): a phase 2, randomised, double-blind, placebo-controlled trial | Repeated trail |
| 3 | Coats, A. J. | 2022 | Patiromer to Manage Hyperkalemia in Patients Receiving Optimized Renin-Angiotensin-Aldosterone System Inhibitors for Heart Failure (DIAMOND): prespecified Analysis of Patients With Hyperkalemia or Previous Hyperkalemia | Repeated trail |
| 4 | Filippatos, G. | 2022 | Patiromer for Hyperkalemia Management in Patients Receiving Renin-Angiotensin-Aldosterone System Inhibitors for Heart Failure (DIAMOND): prespecified Analysis of Patients With or Without Diabetes | Repeated trail |
| 5 | Pitt, B. | 2011 | Evaluation of the efficacy and safety of RLY5016, a polymeric potassium binder, in a double-blind, placebo-controlled study in patients with chronic heart failure (the PEARL-HF) trial | Repeated trail |
| 6 | Pitt, B. | 2010 | The PEARL HF (Multicenter Randomized Double blind Placebo Controlled, Parallel Group Multiple Dose to Evaluate the Effects of RLY5016 in Heart Failure Patients) Trial | Repeated trail |
| 7 | Pitt, B. | 2015 | Patiromer lowers serum potassium and prevents recurrent hyperkalemia in patients with heart failure and CKD when treated with RAAS inhibitors: results from OPAL-HK | Repeated trail |
| 8 | Weir, M. R. | 2015 | Patiromer increased time to RAAS inhibitor discontinuation compared with placebo in advanced CKD patients with hyperkalemia | Repeated trail |
| 9 | Weir, M. R. | 2018 | Effect of Patiromer on Hyperkalemia Recurrence in Older Chronic Kidney Disease Patients Taking RAAS Inhibitors | Repeated trail |
| 10 | Williams, B. | 2019 | Patiromer vs. Placebo to Enable Spironolactone in Patients with Resistant Hypertension and Chronic Kidney Disease (AMBER): results in Prespecified Subgroups | Repeated trail |
| 11 | - | 2022 | Patiromer for hyperkalaemia in heart failure | Review |
| 12 | De Nicola, L. | 2019 | Management of hyperkalemia in Nephrology and Cardiology clinics: reality and perspectives | Review |
| 13 | Gregg, L. P. | 2022 | Steroidal or non-steroidal MRAs: should we still enable RAASi use through K binders? | Review |
| 14 | Kumar, R. | 2017 | Managing hyperkalemia in high-risk patients in long-term care | Review |
| 15 | Weinstein, Jordan | 2022 | Prevention and treatment of hyperkalemia in patients with inhibitors of renine-angiotensin-aldosterone systems | Review |
| 16 | Lizaraso-Soto, F. | 2021 | Binding Potassium to Improve Treatment With Renin-Angiotensin-Aldosterone System Inhibitors: Results From Multiple One-Stage Pairwise and Network Meta-Analyses of Clinical Trials | Review |
| 17 | Maggioni, Aldo P. | 2021 | Prevalence, clinical impact and costs of hyperkalaemia: Special focus on heart failure | Review |
| 18 | Meyer, P. | 2020 | Patiromer and medication optimisation in heart failure with reduced ejection fraction: a Swiss perspective | Review |
| 19 | Pitt, B. | 2018 | The tolerability and safety profile of patiromer: a novel polymer-based potassium binder for the treatment of hyperkalemia | Review |
| 20 | Rakisheva, A. | 2020 | Hyperkalemia in heart failure: Foe or friend? | Review |
| 21 | van der Meer, Peter | 2011 | To bind or not to bind: potassium-lowering drugs in heart failure | Review |
| 22 | Weinstein, Jordan | 2021 | Prevention and management of hyperkalemia in patients treated with renin-angiotensin-aldosterone system inhibitors | Review |
| 23 | Pitt, B. | 2015 | 1-year safety and efficacy of patiromer for hyperkalemia in heart failure patients with chronic kidney disease on reninangiotensin-aldosterone system inhibitors | Not RCT |
| 24 | Pitt, B. | 2018 | Evaluation of an individualized dose titration regimen of patiromer to prevent hyperkalaemia in patients with heart failure and chronic kidney disease | Not RCT |
| 25 | Bakris, G. L. | 2015 | Effect of Patiromer on Serum Potassium Level in Patients With Hyperkalemia and Diabetic Kidney Disease: the AMETHYST-DN Randomized Clinical Trial | No interested outcomes |
| 26 | Bakris, G. L. | 2021 | Hyperkalemia Management in Older Adults With Diabetic Kidney Disease Receiving Renin-Angiotensin-Aldosterone System Inhibitors: a Post Hoc Analysis of the AMETHYST-DN Clinical Trial | No interested outcomes |
| 27 | Kloner, R. A. | 2018 | Effect of Patiromer in Hyperkalemic Patients Taking and Not Taking RAAS Inhibitors | No interested outcomes |
| 28 | Pitt, B. | 2018 | Long-term effects of patiromer for hyperkalaemia treatment in patients with mild heart failure and diabetic nephropathy on angiotensin-converting enzymes/angiotensin receptor blockers: results from AMETHYST-DN | No interested outcomes |
| 29 | Pitt, B. | 2017 | Long-term effects of patiromer for hyperkalaemia treatment in patients with HFrEF and diabetic nephropathy on RAASi | No interested outcomes |
| 30 | - | 2009 | A Multicenter, Randomized, Double-blind, Placebo-Controlled, Parallel-Group, Multiple-Dose Study to Evaluate the Effects of RLY5016 in Heart Failure Patients - PEARL-HF | Study designs or protocols |
| 31 | - | 2017 | A clinical trial evaluating the efficacy of Patiromer in optimizing the therapy with mineralocorticoid receptor antagonists in heart failure patients who also suffer from hyperkalaemia | Study designs or protocols |
| 32 | - | 2009 | Evaluation of Patiromer in Heart Failure Patients | Study designs or protocols |
| 33 | - | 2010 | Evaluation of Patiromer Titration in Heart Failure Patients With Chronic Kidney Disease | Study designs or protocols |

**Table 6** Quality of evidence based on the GRADE framework.

| outcomes | Summary of findings | | Quality assessment | | | | | | Certainty of evidence |
| --- | --- | --- | --- | --- | --- | --- | --- | --- | --- |
|  | No. studies | RR (95%CI) | Risk of bias | Inconsistency | Indirectness | Imprecision | publication bias | Other  consideration |  |
| Incidence of hyperkalemia | 4 | 0.56[0.35,0.87] | serious * | no | no | no | serious † | large effect size | Moederate |
| Tolerance of target dose of MRA | 3 | 1.15[1.01,1.30] | no | no | no | no | no | none | High |
| Discontinuation of RAASi | 3 | 0.48[0.23,1.00] | serious * | no | no | no | serious † | large effect size | Moederate |
